# Insights Into the Adaptation to Greenhouse Cultivation of the Traditional Mediterranean Long Shelf-Life Tomato Carrying the *alc* Mutation: A Multi-Trait Comparison of Landraces, Selections, and Hybrids in Open Field and Greenhouse

**DOI:** 10.3389/fpls.2018.01774

**Published:** 2018-12-04

**Authors:** Maria R. Figàs, Jaime Prohens, María D. Raigón, Leandro Pereira-Dias, Cristina Casanova, María D. García-Martínez, Elena Rosa, Elena Soler, Mariola Plazas, Salvador Soler

**Affiliations:** ^1^Institut de Conservació i Millora de l'Agrodiversitat Valenciana, Universitat Politècnica de València, Valencia, Spain; ^2^Instituto de Biología Molecular y Celular de Plantas, Consejo Superior de Investigaciones Científicas-Universitat Politècnica de València, Valencia, Spain

**Keywords:** breeding, cultivation conditions, fruit quality, genotype × environment interaction, selection, *Solanum lycopersicum*, yield

## Abstract

Long shelf-life tomato (*Solanum lycopersicum*) landraces, characterized by carrying the *alc* allele in the NOR.NAC locus, have been traditionally cultivated in the Mediterranean region. These materials are adapted to open field conditions under low input conditions. However, cultivation under greenhouse is expanding fueled by increasing demand of these traditional tomatoes. We hypothesize that the large diversity in the long shelf-life landraces and derived materials can be exploited for adaptation to these new cultivation conditions. We have evaluated 12 varieties (seven landraces, three selections and two hybrids) carrying the *alc* mutation under open field (OF) and greenhouse (GH) cultivation, and evaluated them for 52 morphological, agronomic, chemical properties, and chemical composition descriptors. All descriptors, except six morphological ones, were variable. The variety effect was the greatest contributor to variation for most morphological traits, as well as for fruit weight, fruit shape, dry matter, and soluble solids content. However, significant environmental and genotype × environment interaction were found for 36 and 42 descriptors, respectively. Fruits from GH plants had lower weight and firmness and were less red than those from OF. On average, in GH yield was 35% lower and daily fruit weight loss in post-harvest 41% higher than in OF. However, fruits from GH had on average higher dry matter and soluble solids contents, antioxidant activity, glucose, fructose, and ascorbic acid concentrations, but lower contents in lycopene and β-carotene than those from OF. A principal components analysis clearly separated varieties according to the cultivation environment. However, the distribution pattern of varieties within each of the two clusters (GH and OF) was similar, despite the strong G × E interaction for many descriptors. Landraces from the same origin plotted in the same area of each cluster, and selections and hybrids plotted together with the landraces. The results reveal a high impact of the cultivation environment on morphological, agronomic, chemical properties, and chemical composition of Mediterranean long shelf-life traditional tomato varieties. This suggests that breeding programs specifically focused to adaptation to greenhouse conditions should be developed.

## Introduction

Tomato (*Solanum lycopersicum* L.) landraces with extremely extended long shelf-life, of several months at room temperature, have been traditionally cultivated in Mediterranean regions (Casals et al., 2012; Bota et al., 2014; Mercati et al., 2015). These landraces are commonly known as “de colgar” in Spanish, “de penjar” or “de ramellet” in Catalan, or “da serbo” in Italian (Bota et al., 2014; Cortés-Olmos et al., 2015; Mercati et al., 2015). These local names make reference to its conservation by hanging in strings (“de colgar” and “de penjar”), to the fact that they normally set in clusters (“de ramellet”), or that have a long storage period (“da serbo”). Before the generalized advent of refrigerators and greenhouse cultivation Mediterranean long shelf-life tomatoes, when stored in ventilated rooms typically hanging in strings with the fruits threaded through the pedicel, allowed the availability of fresh tomatoes throughout the winter time (Casals et al., 2012; Bota et al., 2014; Mercati et al., 2015). This characteristic made its cultivation very popular in several Mediterranean areas, like in the island of Majorca in the first half of the twentieth century (Fairchild, 1927). Despite the general loss of prominence of the Mediterranean long shelf-life tomatoes during the second half of the twentieth century, in the last years there has been an increased interest in these local varieties for their utilization in the traditional local gastronomy (Romero del Castillo et al., 2014). These varieties also are of interest for their resilience and drought tolerance as adaptive traits against climatic change (Maamar et al., 2015; Fullana-Pericàs et al., 2017, 2018).

Several studies reveal that the extended shelf-life of most of the Mediterranean long shelf-life tomatoes of the Spanish “de colgar,” “de penjar,” and “de ramellet” typologies is caused by the *alc* (*alcobaça*) allele of the NAC. NOR gene (Casals et al., 2012; Bota et al., 2014). The *alc* allele also accounts for the long shelf-life of the Italian “da serbo” type (Mercati et al., 2015), but not for other Italian long shelf-life varieties like Corbarino and Lucariello (Tranchida-Lombardo et al., 2018). The *alc* mutation confers a specific phenotype associated to a delayed ripening and reduced lycopene/β-carotene ratio in the fruits (Mutschler et al., 1992; Figàs et al., 2015b), and is found in many different genetic backgrounds (Cebolla-Cornejo et al., 2013). This indicates that throughout the years traditional farmers made an efficient selection of a diverse set of tomato landraces carrying this mutation. As a result, there are many local varieties in the Mediterranean region with the *alc* mutation, with a wide morphological diversity (Cebolla-Cornejo et al., 2013; Bota et al., 2014; Figàs et al., 2015a; Mercati et al., 2015). However, because fruit size in these long shelf-life tomatoes is negatively correlated with the post-harvest conservation period (Casals et al., 2012), fruits are generally smaller than those of standard tomatoes (Bota et al., 2014; Figàs et al., 2015a). Remarkably, these Mediterranean long shelf-life tomatoes use to have a higher dry matter content than standard varieties (Figàs et al., 2015b). The high dry matter content may contribute to its extended post-harvest, and renders them as an interesting material for breeding tomatoes with better flavor (Casals et al., 2011).

The traditional cultivation of the long shelf-life local tomato varieties from the Mediterranean region has been done in the open field with no or reduced irrigation (Mercati et al., 2015; Fullana-Pericàs et al., 2018). The limited availability of water reduced yield dramatically, but improved conservation (Conesa et al., 2014), and decreased the cultivation costs to a minimum, so that even with low yields cultivation was profitable. During the last decades the situation changed completely and modern techniques, including irrigation and increased fertilization have been applied to Mediterranean long shelf-life tomatoes in order to improve yields. In addition, due to increased demand (Romero del Castillo et al., 2014), greenhouse tomato producers started to grow the *alc* traditional long shelf-life tomatoes. Greenhouse cultivation, although more expensive than open field cultivation, allows avoiding costs associated to storage of large quantities of fruits in well-ventilated rooms for long periods. It may also reduce the post-harvest losses due to spoilage of a certain percentage of fruits after months of storage (Casals et al., 2012; Conesa et al., 2014), caused by bruising during harvest and post-harvest handling or due to tomato berries breaking off from the pedicel in fruits hanged on strings. However, these tomato long shelf-life varieties were selected for open field cultivation in the summer season under no or reduced irrigation and low-input conditions (Bota et al., 2014; Mercati et al., 2015; Fullana-Pericàs et al., 2017). As tomato greenhouse conditions involve reduced solar irradiation and high levels of irrigation and fertilization (Peet and Welles, 2005), their adaptation to greenhouse conditions is often suboptimal. Although long shelf-life tomato cultivation has traditionally been based on local landraces (Casals et al., 2012; Bota et al., 2014; Mercati et al., 2015), some local seed companies are marketing selections of this type of tomato and in some cases are producing hybrids with long shelf-life characteristics resulting from the presence of the *alc* mutation (Marín, 2015).

The genetic variation of Mediterranean long shelf-life tomatoes is large (Cebolla-Cornejo et al., 2013; Mercati et al., 2015). Therefore, there are ample opportunities for exploitation of the genotype × environment (G × E) interaction for improving the production and quality of long shelf-life tomatoes under greenhouse. In particular, environmental effects are important for fruit quality, defined by Kyriacou and Rouphael (2018) as “*a dynamic composite of physicochemical properties and evolving consumer perception*,” which embraces organoleptic, nutritional, and bioactive compounds (Hounsome et al., 2008; Barrett et al., 2010; Kaushik et al., 2015). In other works in tomato, G × E interaction in tomato varieties when comparing open field and greenhouse conditions has been very important for both yield and quality traits (Kuti and Konuru, 2005; Roselló et al., 2010; Adalid et al., 2012; Figàs et al., 2018). However, to our knowledge there are no comprehensive evaluations of traits of interest to producers (plant and fruit morphology, agronomic traits), traders (fruit characteristics, post-harvest performance), and consumers (fruit morphology, composition) of a significant number of long shelf-life tomato varieties from different origins and types, including landraces and commercial selections and hybrids.

The aim of the present work is evaluating if the large diversity found among Mediterranean long shelf-life tomatoes carrying the *alc* allele (Cebolla-Cornejo et al., 2013; Bota et al., 2014; Mercati et al., 2015) can be exploited for selecting materials with good adaptation to greenhouse conditions. To test our hypothesis, in this work we evaluate 12 long shelf-life tomato varieties carrying the *alc* mutation from different origins and types (landraces, commercial selections, commercial hybrids) in open field and in greenhouse and characterize them for 52 morphological, agronomic, chemical properties, and chemical composition descriptors. The results obtained will provide relevant information for the enhancement of this varietal type and its adaptation to greenhouse cultivation.

## Materials and Methods

### Plant Materials and Cultivation Conditions

**Figure 1:**
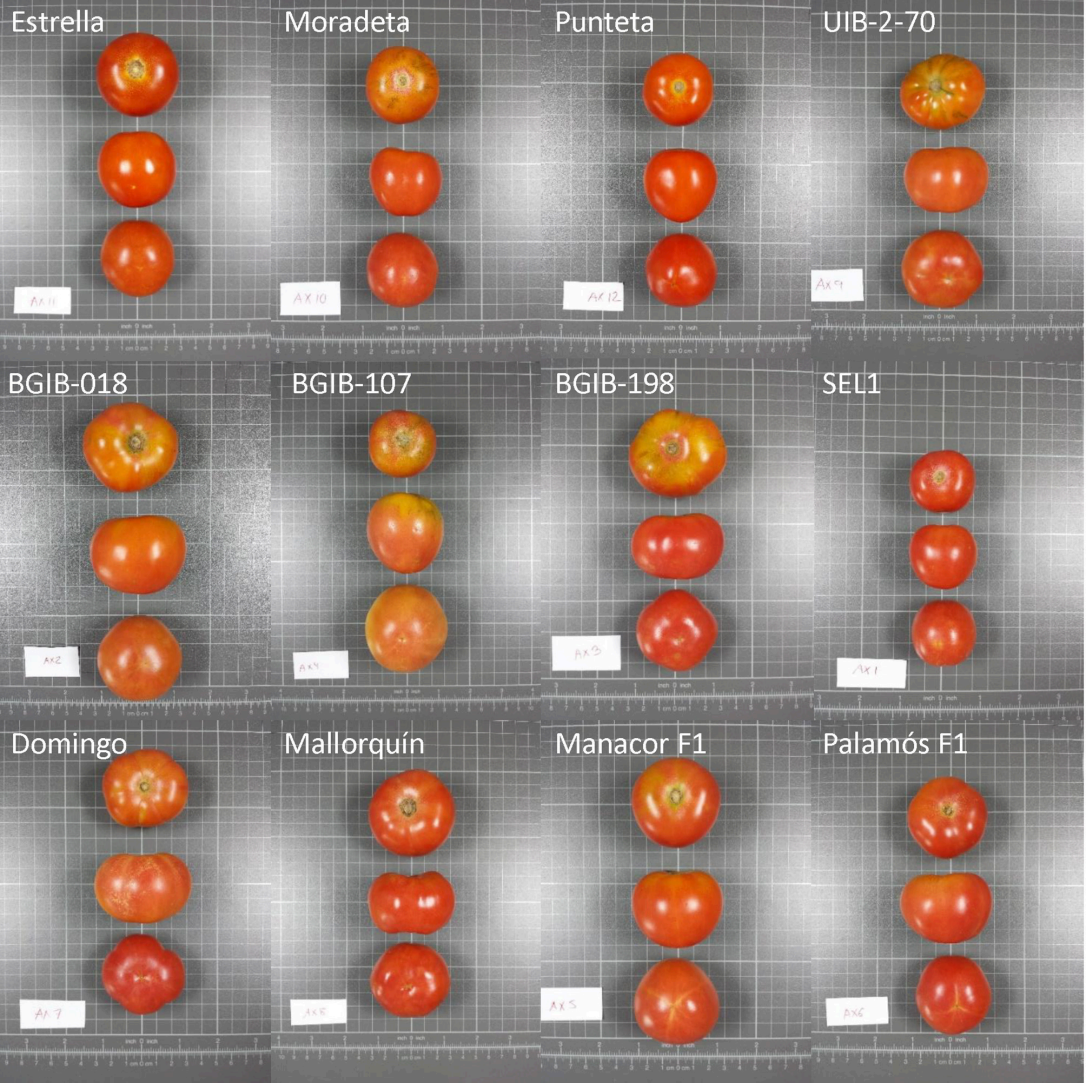
Fruits of the 12 long shelf-life tomato varieties used for the characterization using morphological, agronomic, chemical properties, and chemical composition descriptors. Varieties “Estrella,” “Moradeta,” and “Punteta” correspond to landraces used for the production of the Valencian Community (Spain) Quality Mark “Tomata de Penjar”; variety “UIB-2-70” is the type landrace for the conservation variety “Tomátiga de Ramellet” from Majorca Island (Spain); varieties “BGIB-018,” “BGIB-107,” “BGIB-198” correspond to the “Tomàtiga de Ramellet” highly variable landrace from Majorca Island; variety “SEL1” is a selection used for greenhouse cultivation in the Almería province (Spain), “Domingo,” and “Mallorquín” are commercial varieties corresponding to selections from Semillas Batlle (Molins de Rei, Barcelona, Spain); and “Palamós F1” and “Manacor F1” are two commercial long shelf-life hybrids from Semillas Fitó (Barcelona, Spain). The grid cells in the pictures measure 1 × 1 cm.

Twelve long shelf-life varieties carrying the *alc* allele were used for the present study (Figure 1). Varieties used include: (a) three landraces used for the production of the Valencian Community Quality Mark “Tomata de Penjar” in the Alcalà de Xivert municipality (province of Castellò, mainland Spain) and locally known as “Estrella,” “Moradeta,” and “Punteta”; (b) the type landrace (UIB-2-70) of the conservation variety “Tomátiga de Ramellet” from Majorca Island (Spain); (c) three landraces from the germplasm bank of Universitat de les Illes Balears collected in Majorca Island (BGIB-018, BGIB-107, BGIB-198), corresponding to the “Tomàtiga de Ramellet” highly variable landrace (Bota et al., 2014); (d) a selection of long shelf-life (*alc*) tomato used for greenhouse cultivation in the Almería province (Spain) called “SEL1”; (e) two commercial varieties corresponding to selections of the long shelf-life (*alc*) tomato type (“Domingo” and “Mallorquín”) from Semillas Batlle (Molins de Rei, Barcelona, Spain); and (f) two commercial long shelf-life hybrids (“Palamós F1” and “Manacor F1”) both of which are resistant to *Tomato mosaic virus* (ToMV) and to *Tomato spotted wilt virus* (TSWV), and also to *Fusarium oxysporum* f. sp. *lycopersici* in the case of “Manacor F1,” from Semillas Fitó (Barcelona, Spain).

**Figure 2:**
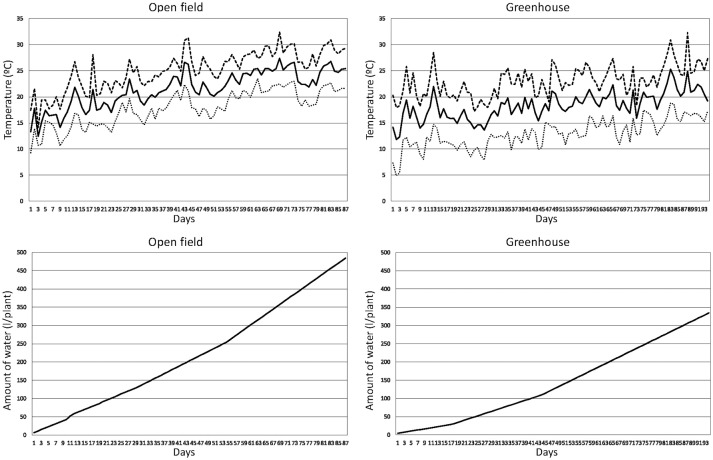
Time course of temperatures and accumulated amounts of water per plant for the open field and greenhouse experiments with 12 long shelf-life tomato varieties. The graphs represent the daily minimum (dotted line), maximum (dashed line), and average (solid line) temperatures since the start of the experiments, which were the 29 April 2016 (open field experiment) and 19 February 2016 (greenhouse experiment), as well as the accumulated amounts of water per plant provided through the irrigation system (plus the rainfall water in the open field experiment).

The 12 varieties were grown under both open field (OF) and greenhouse (GH), with 10 plants per variety under each of the conditions. Plants in each condition were distributed in a completely randomized design, making a total of 10 replicates with one plant per replicate. Prior to germination, seeds were disinfected with a 1:10 w/v solution of dodecahydrate trisodium phosphate (Na_3_PO_4_·12H_2_O) for 3 h and rinsed three times with distilled water; after that a new round of disinfection was performed with a solution of 0.37% sodium hypochlorite (NaOCl) for 1 h followed by three rinsings of 10 min with distilled water. After that, seeds were left to dry on filter paper for several days under room conditions and then placed in hermetic flasks with dry silica gel for several weeks. After that, seeds were thermotreated at 80°C for 24 h. Disinfected seeds were germinated in commercial substrate seedling trays and transplanted when plantlets had a height of around 12–15 cm. Transplanting of OF and GH trials was performed on 29 April 2016 and 19 February 2016, respectively, and lasted until 27 July 2016 and 25 May 2016. These are typical growing cycles in open field and greenhouse cultivation in the area, and dates used for the transplant are within the usual ranges used by commercial farmers. Minimum, maximum, and average temperatures throughout the cultivation period in OF were, respectively, of 9.3, 31.9, and 22.4°C, while in GH were of 4.9, 32.3, and 18.3°C, respectively. The time course of minimum, maximum and average temperatures throughout the cultivation period is presented in Figure 2. The soils of both environments were of the USDA clay-loam soil texture class, with an organic matter of content of 2.72% in OF and 2.64% in GH and a pH of 7.92 in OF and 7.99 in GH.

The open field plot was located in Alcalà de Xivert (Castelló, Spain) in the area of traditional cultivation of the Quality Mark “Tomata de Penjar.” Plants were spaced 0.70 m among rows and 0.50 m within rows. The traditional cultivation techniques were performed, and plants were staked with canes and left unpruned. Irrigation was provided through a drip irrigation system depending on the needs of the plant for a total volume of 1356.7 mm, to which 28.4 mm of pluviometry have to be added, making a total of 484.8 l/plant (Figure 2). After an initial watering of 6.5 l/plant just after the transplant, 4 l/plant were applied daily until the day 29 after transplant, followed by 5 l/plant per day until the day 53 after transplant, and finally 7 l/plant per day every 2 days until the end of the experiment (day 87 after transplant). Pluviometry was mostly concentrated on days 11 (14.4 mm), 12 (5.6 mm), 20 (3.6 mm), and 24 (2.8 mm), while the rest was scattered in seven different non-consecutive days with a range between 0.2 and 0.4 mm per day. A background fertilization consisting of 2.85 kg/m^2^ of poultry manure (2.4% N, 1.0% P, and 1.2% K) was applied before transplant. A top-dressing fertilization of 0.042 kg/m^2^ of fertilizer containing 19% N, 6% P_2_O_5_, and 6% K_2_O was applied 2 weeks after transplant and 0.042 kg/m^2^ of fertilizer containing 15% N, 5% P_2_O_5_, and 30% K_2_O was applied after fruits of the second or third trusses were in the cell expansion fruit development stage. For the OF trial, this makes an average amount of N, P, and K macroelements supplied with fertilizers of 28.9 g/plant of N, 11.6 g/plant of P, and 17.3 g/plant of K.

The plastic greenhouse used for the evaluation was also located in Alcalà de Xivert at a distance of 3 km from the open field plot. The greenhouse was of the multispan type and each module had a size of 52 × 8 m. The ceiling and laterals were covered with Celloflex 4 TT (Riviera Blumen, Puerto Lumbreras, Spain) multilayer polyethylene plastic. This greenhouse had automated cenital openings as well as manually operated lateral openings. Plants were distributed using the same plant density than for OF. Plants were trellised using vertical strings, pruned to remove side shoots. Irrigation was provided using a drip irrigation system like for OF using a total volume of 955.7 mm (334.5 l/plant; Figure 2). After an initial watering of 4.5 l/plant just after the transplant, 1.5 l/plant were applied daily until the day 17 after transplant, followed by 3 l/plant per day days until the day 43 after transplant, and finally 4.5 l/plant per day until the day 93 (1 day before the end of the experiment). The fertilization was the same than for OF, except that poultry manure was applied at a rate of 2.00 kg/m^2^. For the GH trial, this makes an average amount of N, P, and K macroelements supplied with fertilizers of 21.8 g/plant of N, 8.6 g/plant of P, and 13.7 g/plant of K.

Preventive phytosanitary treatments were performed against whiteflies and *Tuta absoluta* with imidacloprid and emamectin in both OF and GH conditions, and weeds were removed with a hoe. Fruits of both trials were harvested at the red maturity stage (i.e., when fruits have between 60 and 90% of the skin with the typical fully ripe color of each accession) according to the scale defined by Yamaguchi (1983). Fruits used for morphometric and chemical measurements and analyses were harvested individually in a single day for each of the accessions when sufficient amounts of fruits at the appropriate red maturity stage were available in the plants.

### Characterization

Varieties were characterized using 52 descriptors, including morphological (34), agronomic (6), physico-chemical properties (6), and chemical composition (6) traits. Morphological and agronomic descriptors were measured on a plant basis (*n* = 10). The morphological descriptors were quantitative (6), meristic (2), based a quantitative scale (19), or dichotomic (7) and corresponded to IPGRI (1996) tomato characterization descriptors (Table 1). The agronomic descriptors considered were: fruit weight (g); fruit shape (ratio length/width) obtained from IPGRI descriptors Fruit length and Fruit width; fruit firmness (Shore A standard units) measured in two opposite sides in the mid-part of the fruit between the proximal and distal ends of the fruit using a 53215 Fruit Hardness Tester (TR Turoni srl, Forli, Italy); color difference with true red obtained using the formula [(L^*^-50)^2^+(a^*^-60)^2^+b^*2^]^0.5^ from CIELAB fruit color parameters L^*^, a^*^, and b^*^ measured in the central part of the fruit at a mid-distance between the distal and proximal parts using a CR-300 (Minolta, Osaka, Japan) chromameter; yield (kg/plant); and, daily moisture loss (%) by measuring the fruit weight at harvest and after storage for 30 days at room temperature of a sample of 10 fruits per plant and calculating the average daily loss. For descriptors involving measurements of fruits, 10 fruits per plant were measured and values obtained for individual fruits were used to calculate the average value for each individual plant.

**Table 1:** Morphological descriptors used for the characterization of 12 long shelf-life tomato varieties in two environments. Full details of the descriptors used can be consulted elsewhere (IPGRI, 1996).

**Descriptors**	**IPGRI descriptor code**	**Units/scale**
Plant growth type	7.1.2.1	1 = Dwarf; 4 = Indeterminate
Plant size	7.1.2.2	3 = Small; 7 = Large
Stem pubescence intensity	7.1.2.4	3 = Sparse; 7 = Dense
Foliage density	7.1.2.6	3 = Sparse; 7 = Dense
Number of leaves under 1st inflorescence	7.1.2.7	–
Leaf attitude	7.1.2.8	3 = Semi-erect; 7 = Dropping
Degree of leaf dissection	7.1.2.10	3 = Low; 7 = High
Anthocyanin coloration of leaf veins	7.1.2.11	1 = Obscure vein; 2 = Clear (normal)
Inflorescence type	7.2.1.1	1 = Generally uniparous; 3 = Generally multiparous
Number of flowers per inflorescence	8.1.5	–
Corolla blossom type	7.2.1.3	1 = Closed; 2 = Open
Style position	7.2.1.7	1 = Inserted; 4 = Highly exserted
Style shape	7.2.1.8	1 = Simple; 3 = Divided
Style hairiness	7.2.1.9	0 = Absent; 1 = Present
Dehiscence	7.2.1.11	1 = Poricidal; 2 = Longitudinal
Exterior color of immature fruit	7.2.2.1	1 = Greenish-white; 9 = Very dark green
Presence of green (shoulder) trips on the fruit	7.2.2.2	0 = Absent; 1 = Present
Intensity of greenback (shoulder)	7.2.2.3	3 = Slight; 7 = Strong
Fruit pubescence	7.2.2.4	3 = Sparse; 7 = Dense
Fruit size homogeneity	7.2.2.7	3 = Low; 7 = High
Fruit length	7.2.2.9	mm
Fruit width	7.2.2.10	mm
Easiness of fruit to detach from pedicel	7.2.2.15	3 = Easy; 7 = Difficult
Fruit shoulder shape	7.2.2.16	1 = Flat; 7 = Strongly depressed
Pedicel length	7.2.2.17	cm
Pedicel length from abscission layer	7.2.2.18	cm
Presence/absence of jointless pedicel	7.2.2.19	0 = Absent; 1 = Present
Width of pedicel scar	7.2.2.20	mm
Size of corky area around pedicel scar	7.2.2.21	mm
Skin color of ripe fruit	7.2.2.23	1 = Colorless; 2 = Yellow
Fruit blossom end shape	7.2.2.33	1 = Indented; 3 = Pointed
Radial cracking^a^	8.2.3	1 = Corky lines; 7 = Severe
Concentric cracking^a^	8.2.4	1 = Corky lines; 7 = Severe
Fruit fasciation	8.2.5	3 = Slight; 7 = Severe

a *Values of 0 were assigned for these descriptors when cracking was not observed*.

The chemical properties and chemical composition descriptors were measured on six samples (*n* = 6) taken from the bulked harvest of all plants, with at least five fruits per sample. Samples were squeezed with a domestic juice extractor and two aliquots were obtained: one for immediate determination of several traits and another one was frozen in liquid N_2_ and stored at −80°C until used for the other traits.

Chemical properties measured were: dry matter (%) by drying at 105°C until constant weight; soluble solids (SS; %) by refractometry; pH with a pHmeter; titratable acid (TA; %) by titration of diluted juice (1:5) with 0.5 N NaOH to pH 8.1 and expressed as citric acid percentage; taste index (TI) by applying the formula TI = TA + (SS/(20 × TA)) according to Navez et al. (1999); and, antioxidant activity (mM TE/g), measured using the colourimetric DPPH assay and expressed as Trolox equivalents (TE). All chemical properties were determined in the immediate analysis aliquot, with the exception of antioxidant activity, which was measured in the frozen aliquot. Chemical composition traits evaluated were the contents in: glucose (g/kg) and fructose (g/kg) measured using the D-Fructose/D-Glucose Assay Kit (Megazyme International Ltd., Wicklow, Ireland); citric acid using the CI9920 enzymatic kit (BEN S.r.l., Milan, Italy); ascorbic acid (mg/kg) by potentiometric titration with a Titrino 702 (Metrohm, Herisau, Switzerland) potentiometric titrator using a Metrohm 6.0420.100 combined Pt selective electrode and a 0.005 M chloramine solution as standard; lycopene (mg/kg) and β-carotene (mg/kg) by extraction overnight in darkness with ethanol:hexane (4:3 v/v), followed by separation of the hexane phase and determination of lycopene and β-carotene by UV/V spectrophotometry absorbance at 503 nm (lycopene) and 450 nm (β-carotene). All chemical composition analysis were performed in the frozen aliquot homogenate, except ascorbic acid, which was measured in the aliquot used for immediate analysis. Full details of the procedures for determining chemical properties and chemical composition traits are described elsewhere (Figàs et al., 2015b).

### Data Analyses

Data for the morphological, agronomic, chemical properties, and chemical composition descriptors were subjected to a two factorial (variety and environment) analysis of variance (ANOVA) including the interaction among both main factors. The total sums of squares was partitioned in the sums of squares for variety, environment, variety × environment, and residual effects. For morphological descriptors, means and range were obtained for each environment. For agronomic, chemical properties, and chemical composition descriptors, the average value for each variety in each environment was calculated and the average standard error (SE) was obtained from the ANOVA analyses. Significance of differences among variety × environment combinations was evaluated using a Student-Newman-Keuls multiple range test at *P* = 0.05. A principal components analysis (PCA) was performed using pairwise Euclidean distances among variety means for each environment using standardized data (μ = 0; σ = 1) for the descriptors that were variable. All statistical analyses were performed using the Statgraphics Centurion XVI (StatPoint Technologies, Warrenton, VA, USA) software.

## Results

### Analysis of Variance

Out of the 52 descriptors evaluated, six morphological descriptors were uniform across all varieties and environments. These descriptors and their states were: Corolla blossom type (1 = Closed); Style shape (1 = Simple); Dehiscence (2 = Longitudinal); Fruit pubescence (3 = Sparse); Presence/absence of jointless pedicel (0 = Absent); Concentric cracking (0 = No cracking). For the rest of descriptors, significant differences (*P* < 0.05) were found among varieties (Table 2). The percentage of sums of squares accounted for by the variety effect ranged between 8.8% (Radial cracking) and 100% (for Plant growth type and Skin color of ripe fruit). The variety effect was the greatest contributor to the sums of squares for most morphological descriptors. However, for the rest of descriptors the variety effect was only the greatest contributor for the agronomic descriptors Fruit weight and Fruit shape and for the chemical properties descriptors Dry matter and Soluble solids (Table 2). Significant differences among cultivation environments were found for 36 out of the 46 variable descriptors. Traits non-significant for the cultivation environment effect were six morphological ones as well as four related to chemical properties and composition (mostly related to acidity). The environmental effect was the main contributor to the sums of squares only for five descriptors, of which four were morphological (Foliage density, Leaf attitude, Pedicel length, and Width of pedicel scar) and the other one was the chemical composition descriptor Glucose (Table 2). The variety × environment interaction effect was significant for all variable descriptors, except for four (Plant growth type, Skin color of ripe fruit, pH, and Ascorbic acid). The variety × environment interaction was the greatest contributor to the sums of squares for three morphological descriptors (Number of leaves under 1st inflorescence, Intensity of greenback, and Fruit fasciation), while it had the same contribution than Variety for five other morphological descriptors (Table 2). The residual effect was the greatest contributor to the sums of squares for 14 descriptors, of which two were morphological (Pedicel length from abscission layer, and Radial cracking), four agronomic (all except Fruit weight and Fruit shape), three chemical properties (pH, Titratable acidity, and Antioxidant activity), and five chemical composition (all except Glucose) descriptors.

**Table 2:** Percentage of the total sums of squares for the effects of variety, environment, interaction between variety, and environment and residuals.

	**Sums of squares**^**a**^			
**Descriptors**	**Variety**	**Environment**	**Variety ×** **environment**	**Residual**
**MORPHOLOGICAL DESCRIPTORS**				
Plant growth type	100.0^***^	0.0^ns^	0.0^ns^	0.0
Plant size	54.2^***^	24.1^***^	20.8^***^	0.9
Stem pubescence density	48.9^***^	2.1^***^	48.9^***^	0.0
Foliage density	22.8^***^	46.3^***^	30.9^***^	0.0
Number of leaves under 1st inflorescence	29.3^***^	0.2^ns^	42.9^***^	27.5
Leaf attitude	21.4^***^	55.2^***^	21.4^***^	2.0
Degree of leaf dissection	43.4^***^	17.8^***^	38.3^***^	0.5
Anthocyanin coloration of leaf veins	47.8^***^	4.3^***^	47.8^***^	0.0
Inflorescence type	37.6^***^	21.8^***^	11.6^***^	29.0
Number of flowers per inflorescence	47.9^***^	12.8^***^	11.0^***^	28.4
Style position	57.4^***^	6.2^***^	14.9^***^	21.4
Style hairiness	42.9^***^	14.3^***^	42.9^***^	0.0
Exterior color of immature fruit	45.5^***^	5.0^***^	45.5^***^	4.0
Presence of green (shoulder) trips on the fruit	47.8^***^	4.3^***^	47.8^***^	0.0
Intensity of greenback (green shoulder)	40.0^***^	0.0^ns^	58.1^***^	1.9
Fruit size homogeneity	39.9^***^	4.8^***^	38.1^***^	17.2
Fruit length	67.7^***^	0.1^ns^	6.7^***^	25.5
Fruit width	59.6^***^	14.3^***^	6.8^***^	19.4
Easiness of fruit to detach from pedicel	49.5^***^	33.6^***^	16.8^***^	0.0
Fruit shoulder shape	46.5^***^	33.2^***^	7.4^***^	13.0
Pedicel length	15.9^***^	42.6^***^	7.4^***^	34.1
Pedicel length from abscission layer	49.2^***^	8.5^***^	8.3^***^	34.0
Width of pedicel scar	24.0^***^	42.6^***^	15.2^***^	18.2
Size of corky area around pedicel scar	23.7^***^	20.8^***^	13.6^***^	41.8
Skin color of ripe fruit	100.0^***^	0.0^ns^	0.0^ns^	0.0
Fruit blossom end shape	61.9^***^	5.1^***^	6.7^***^	26.3
Radial cracking	8.8^*^	0.6^ns^	9.3^*^	81.4
Fruit fasciation	43.0^***^	2.4^***^	48.2^***^	6.4
**AGRONOMIC DESCRIPTORS**				
Fruit weight (g)	50.4^***^	22.4^***^	9.9^***^	17.3
Fruit shape	68.1^***^	12.9^***^	2.3^*^	16.7
Fruit firmness (Shore A standard units)	33.4^***^	12.0^***^	15.2^***^	39.4
Color difference with true red	24.2^***^	24.1^***^	6.5^**^	45.3
Yield (kg/plant)	18.0^***^	16.4^***^	12.2^***^	53.4
Daily moisture loss (%)	24.2^***^	24.1^***^	6.5^**^	45.3
**CHEMICAL PROPERTIES DESCRIPTORS**				
Dry matter (%)	49.3^***^	9.0^***^	15.8^***^	25.9
Soluble solids (%)	53.3^***^	2.1^**^	13.8^***^	30.9
pH	42.8^***^	1.2^ns^	4.3^ns^	51.6
Titratable acidity (%)	34.9^***^	0.1^ns^	10.8^*^	54.3
Taste index	65.1^***^	0.1^ns^	8.7^***^	26.1
Antioxidant activity (mM TE/g)	17.7^***^	8.3^***^	14.7^**^	59.3
**CHEMICAL COMPOSITION DESCRIPTORS**				
Glucose (g/kg)	11.3^***^	38.9^***^	12.9^***^	36.9
Fructose (g/kg)	17.6^***^	9.9^***^	12.1^*^	60.4
Citric acid (g/kg)	26.8^***^	0.0^ns^	18.5^***^	54.7
Ascorbic acid (mg/kg)	40.3^***^	3.6^**^	5.1^ns^	51.0
Lycopene (mg/kg)	16.0^**^	10.5^***^	14.2^***^	59.3
β-carotene (mg/kg)	13.0^*^	2.5^*^	18.3^**^	66.2

*Descriptors include 46 morphological, agronomic, chemical properties, and chemical composition descriptors evaluated for which variation was observed in 12 long shelf-life tomato varieties grown in two environments (open field and greenhouse)*.

a, ns, *, **, and *** *Non-significant, or significant at p < 0.05, p < 0.01, and p < 0.001, respectively*.

### Variation for Morphological Descriptors

A wide range of variation among accessions for the 28 variable morphological descriptors was observed both under OF and GH environments (Table 3). For traits measured in a quantitative scale in most cases the range of variation covered an important part of the scale range. An exception was the Radial cracking in which a narrow range of variation was observed for this descriptor, as the incidence of cracking was very low (Table 3). For quantitative and meristic descriptors, a considerable variation was also observed, with differences of over four-fold for the Number of flowers per inflorescence.

**Table 3:** Means and range of variation for varietal means for 28 variable morphological descriptors in 12 long shelf-life tomato varieties grown in two environments (open field and greenhouse).

	**Open field**		**Greenhouse**	
**Descriptors**	**Mean**	**Range**	**Mean^a^**	**Range**
Plant growth type	3.83	2–4	3.83^ns^	2–4
Plant size	6.50	5–7	5.58^***^	3–7
Stem pubescence density	5.00	5–5	4.83^***^	3–6
Foliage density	6.58	4–7	4.58^***^	3–7
Number of leaves under 1st inflorescence	6.99	6.3–7.3	7.13^ns^	4.5–9.3
Leaf attitude	5.00	5–5	6.05^***^	5–7
Degree of leaf dissection	5.49	5–6	4.75^***^	3–6
Anthocyanin coloration of leaf veins	1.92	1–2	2.00^***^	2–2
Inflorescence type	1.45	1–2.5	2.16^***^	1–3
Number of flowers per inflorescence	5.99	4.5–11.2	8.32^***^	3.5–15.1
Style position	2.00	1.1–3.7	1.67^***^	1.0–2.4
Style hairiness	1.00	1–1	0.75^***^	0–1
Exterior color of immature fruit	3.18	3–5	3.00^***^	3–3
Presence of green (shoulder) trips on the fruit	0.92	0–1	1.00^***^	1–1
Intensity of greenback (green shoulder)	3.93	3–5	3.92^ns^	3–6
Fruit size homogeneity	6.09	5–7	5.83^***^	5–6
Fruit length	4.33	3.70–4.96	4.30^ns^	3.50–4.91
Fruit width	5.65	4.70–7.34	5.08^***^	4.08–5.87
Easiness of fruit to detach from pedicel	4.42	3–5	3.42^***^	3–5
Fruit shoulder shape	3.68	1.4–5	2.28^***^	1–3
Pedicel length	2.44	2.05–3.19	3.32^***^	2.92–3.88
Pedicel length from abscission layer	0.81	0.70–1.05	0.90^***^	0.70–1.04
Width of pedicel scar	0.72	0.48–1.23	0.41^***^	0.30–0.55
Size of corky area around pedicel scar	0.13	0.10–0.22	0.20^***^	0.08–0.30
Skin color of ripe fruit	1.17	1–2	1.17^ns^	1–2
Fruit blossom end shape	1.41	1–3	1.70^***^	1–3
Radial cracking	0.01	0–0.1	0.05^ns^	0–0.6
Fruit fasciation	0.10	0–0.7	0.36^***^	0–4

*Units or scale for each descriptor can be consulted in Table 1*.

ans, and *** *Differences between open field and greenhouse environments are non-significant, or significant at p < 0.001, respectively*.

GH cultivation conditions resulted in relevant changes in the plant morphology compared to OF conditions, although the ranges of variation overlapped for all descriptors (Table 3). If we consider morphological traits for which there is a change of over 10% in GH with respect to OF, plants grown in GH had smaller plant size, less foliage density, leaves with a greater degree of dropping, and less divided, inflorescences with higher division and with more flowers, less exerted, and hairy styles, fruits less wide and easier to detach from pedicel, flatter fruit shoulder shape, longer pedicels, smaller pedicel scar, greater corky area around the pedicel scar, more pointed, and with greater fruit fasciation than those from OF (Table 3).

### Variation for Agronomic Descriptors

Fruits from OF had a greater fruit weight (on average 40%) than those from GH, with a considerable variation among varieties, ranging between 60.2 g/fruit (“Moradeta”) to 161.0 g/fruit (“Mallorquín”) for OF and between 51.2 g/fruit (“Moradeta”) and 89.0 g/fruit (“BGIB-018”) for GH (Table 4). Fruits from OF were more flattened than those of GH, although under both conditions all varieties had a fruit length/width ratio below 1, except for variety “Punteta,” with a value of 1.015 under GH conditions. Fruits from OF conditions were more firm than those of GH, with the exception of variety “SEL1” (Table 4). A smaller range of variation was observed for OF (between 46.8 Shore A standard units for “BGIB-018” and 67.4 Shore A standard units for “Punteta”) than for GH (between 31.7 Shore A standard units for “Estrella” and 59.5 Shore A standard units for “Manacor F1”). Color difference with true red was of lower magnitude and the range of variation was narrower under OF than under GH (Table 4). Yield was, on average, 35% higher under OF than under GH. All varieties had a higher yield under OF than under GH, with the exception of “Domingo.” Considerable variation among varieties was observed with ranges of variation between 2.92 kg/plant for “SEL1” and 5.03 kg/plant for “BGIB-107” in OF and between 1.84 kg/plant for “Punteta” and 4.0 kg/plant of “Domingo” in GH (Table 4). Fruits from GH had a higher (on average 41%) moisture loss during post-harvest than those from OF. Under both conditions the variety with lower daily moisture loss was “BGIB-107” with values of 0.175% and 0.243% under OF and GH, respectively, while the one with higher moisture loss was “Estrella” with values of 0.348 and 0.476% under OF and GH, respectively (Table 4).

**Table 4:** Mean values for agronomic descriptors for 12 long shelf-life tomato varieties grown in open field (OF) and greenhouse (GH) environments.

	**Fruit weight (g)**^**a**^		**Fruit shape** **(length/width ratio)**		**Fruit firmness** **(Shore a standard units)**		**Color difference with true red**		**Yield** **(kg/plant)**		**Daily moisture** **loss (%)**	
**Variety**	**OF**	**GH**	**OF**	**GH**	**OF**	**GH**	**OF**	**GH**	**OF**	**GH**	**OF**	**GH**
BGIB-018	97.9 hij	89.0 fgh	0.736 bcd	0.757 b–f	46.8 b–e	48.0 b–e	48.4 hi	45.8 e–h	4.58 fgh	3.05 a–e	0.183 a	0.286 a–f
BGIB-107	105.7 ijk	71.5 cde	0.876 ij	0.973 kl	60.9 hi	55.4 e–h	47.3 f–i	46.8 f–i	5.03 h	2.31 ab	0.175 a	0.243 a–e
BGIB-198	114.9 k	71.6 cde	0.688 ab	0.778 c–g	53.2 b–h	49.8 b–f	45.3 d–h	44.1 c–f	4.17 e–h	2.59 a–d	0.223 abc	0.404 g–j
Domingo	73.1 c–f	51.2 ab	0.667 a	0.717 abc	59.7 ghi	45.4 bc	49.7 i	45.8 e–h	3.47 b–g	4.30 e–h	0.196 ab	0.359 e–i
Estrella	84.4 e–h	59.6 bcd	0.788 d–g	0.846 ghi	54.8 d–h	31.7 a	46.9 f–i	47.2 f–i	4.32 e–h	2.44 abc	0.348 d–i	0.476 j
Mallorquín	161.0 l	87.1 e–h	0.657 a	0.803 d–h	52.3 b–h	44.4 b	44.7 c–g	42.1 bcd	4.77 gh	3.84 c–h	0.245 a–e	0.423 hij
Manacor F1	108.9 jk	75.7 d–g	0.872 hij	0.917 jk	59.6 ghi	59.5 ghi	48.0 ghi	46.8 f–i	4.63 fgh	3.91 d–h	0.275 a–e	0.443 ij
Moradeta	60.2 bcd	51.2 ab	0.827 f–i	0.915 jk	60.4 ghi	45.9 bcd	42.2 bcd	42.0 bc	3.74 b–h	2.85 a–e	0.304 b–g	0.334 c–h
Palamós F1	91.5 ghi	76.0 d–g	0.750 b–e	0.822 e–i	60.0 ghi	57.1 fgh	47.4 f–i	45.8 e–h	4.16 e–h	2.97 a–e	0.226 abc	0.391 f–j
Punteta	65.9 bcd	41.1 a	0.962 kl	1.015 l	67.4 i	58.2 fgh	40.7 b	37.4 a	3.15 a–e	1.84 a	0.302 b–g	0.323 c–h
SEL1	58.1 bc	52.4 ab	0.813 e–i	0.920 jk	49.7 b–f	54.0 c–h	42.9 b–e	42.2 bcd	2.92 a–e	2.32 ab	0.235 a–d	0.307 b–g
UIB-2-70	100.2 hij	73.3 c–f	0.694 ab	0.791 d–g	51.3 b–e	47.5 b–g	46.3 fgh	46.2 fgh	3.09 a–f	3.27 a–e	0.201 ab	0.255 a–e
SE	3.93		0.017		2.0		0.7		0.31		0.025	

*The significance of the effects Variety, Environment (OF vs. GH), and Variety × Environment are presented in Table 2. The standard error (SE) for pairwise comparison of the 24 combinations of Variety and Environment is provided*.

a *For each trait, mean values for combinations of Variety, and Environment separated by different letters are significant (P < 0.05) according to the Student-Newman-Keuls multiple range test. When a mean is followed by four or more letters, the range of letters is indicated*.

### Variation for Chemical Properties Descriptors

On average, fruits from GH cultivation had higher dry matter content (8.5%) than those from OF conditions, although for four varieties values were higher under OF conditions (Table 5). Values ranged between 5.61% for “BGIB-107” and 8.04% for “SEL1” under OF and between 5.46% for “BGIB-107” and 8.76% for “Punteta” under GH. Similarly, for soluble solids content fruits from GH had higher average contents than those of OF, although the differences were smaller (3.7%) than for dry matter content, and in five varieties the contents under OF were higher than those of GH (Table 5). As occurred for dry matter content, the variety with lowest values was “BGIB-107” with 5.23 and 5.05% under OF and GH, respectively, while the ones with highest values were “SEL1” under OF (6.98%) and “Moradeta” under GH (7.92%). Regarding pH, average differences among environments were non-significant, although for some varieties significant differences existed among environments (Table 5). The variety with lowest pH values was “BGIB-198” (4.08 in both environments) and the ones with highest values were “Estrella,” “Mallorquín,” and “Moradeta” under OF (4.38) and the latter under GH (4.39). As for pH, no significant differences were observed among environments for titratable acidity, although a considerable range of variation within each environment was observed, with values between 0.37% for “Mallorquín” and 0.62% for “BGIB-018” under OF and between 0.40% for “Estrella” and 0.58% for “Moradeta” under GH (Table 5). No differences among environments were observed for taste index among environments, although for some varieties significant differences were observed. In all cases the taste index value was above 1, with the lowest values observed in “BGIB-107” (1.02 in both environments) and the highest in “Punteta” (1.28 in OF and 1.32 in GH) (Table 5). The antioxidant activity was higher under GH (on average 25.6%) than under OF, although for three varieties it was higher under OF (Table 5). A wide range of variation was observed for antioxidant activity among varieties in both conditions, with values ranging between 0.57 mM TE/g for “Estrella” and 1.26 mM TE/g for “Palamós F1” under OF, and between 0.66 mM TE/g for “BGIB-018” and 1.70 mM TE/g for “Domingo” under GH (Table 5).

**Table 5:** Mean values for chemical properties descriptors for 12 long shelf-life tomato varieties grown in open field (OF) and greenhouse (GH) environments.

	**Dry matter (%)**^**a**^		**Soluble solids (%)**		**pH**		**Titratable acidity (%)**		**Taste index**		**Antioxidant activity** **(mM TE/g)**	
**Variety**	**OF**	**GH**	**OF**	**GH**	**OF**	**GH**	**OF**	**GH**	**OF**	**GH**	**OF**	**GH**
BGIB-018	7.34 d–h	6.78 c–f	6.58 ef	5.83 bcd	4.17 a–d	4.18 a–d	0.62 ef	0.50 a–f	1.18 cde	1.08 ab	1.12 abc	0.66 ab
BGIB-107	5.61 ab	5.46 a	5.23 ab	5.05 a	4.15 abc	4.18 a–d	0.50 a–f	0.44 a–e	1.02 a	1.02 a	0.77 ab	0.76 ab
BGIB-198	6.30 bc	8.51 ij	6.13 def	6.68 ef	4.08 a	4.08 a	0.60 def	0.65 f	1.11 bc	1.17 b–e	0.72 ab	1.01 ab
Domingo	6.19 abc	7.40 d–h	5.52 abc	6.35 def	4.26 a–d	4.32 bcd	0.42 abc	0.49 a–f	1.08 ab	1.14 bcd	0.89 ab	1.70 c
Estrella	7.16 c–h	7.08 c–g	6.37 def	5.97 bcd	4.38 cd	4.26 a–d	0.42 abc	0.40 ab	1.21 def	1.15 b–e	0.57 a	1.09 abc
Mallorquín	6.40 bcd	7.03 c–g	5.93 bcd	6.23 def	4.38 cd	4.33 bcd	0.37 a	0.45 a–e	1.17 b–e	1.15 b–e	0.81 ab	0.90 ab
Manacor F1	7.43 d–h	7.80 f–j	6.75 ef	6.72 ef	4.24 a–d	4.21 a–d	0.49 a–f	0.50 a–f	1.23 d–g	1.17 b–e	0.97 ab	1.18 abc
Moradeta	7.84 f–j	8.76 j	6.58 ef	7.92 h	4.38 cd	4.39 d	0.43 a–d	0.49 a–f	1.24 efg	1.30 gh	0.82 ab	1.18 abc
Palamós F1	6.65 cde	8.17 hij	6.27 def	6.50 ef	4.25 a–d	4.11 ab	0.48 a–f	0.58 b–f	1.14 bcd	1.14 bcd	1.26 abc	1.38 bc
Punteta	7.47 e–h	8.57 ij	6.78 ef	7.82 h	4.34 cd	4.33 bcd	0.40 a	0.45 a–e	1.28 fgh	1.32 h	0.72 ab	1.30 abc
SEL1	8.04 g–j	7.75 f–i	6.98 f	7.00 f	4.30 a–d	4.25 a–d	0.55 c–f	0.45 a–e	1.22 d–g	1.23 d–g	1.18 abc	1.15 abc
UIB-2-70	6.81 c–f	7.12 c–g	6.32 def	6.13 def	4.18 a–d	4.10 a	0.60	0.54	1.17	1.11 bc	0.91 ab	1.29 abc
SE	0.23		0.20		0.05		0.04		0.02		0.14	

*The significance of the effects Variety, Environment (OF vs. GH), and Variety × Environment are presented in Table 2. The standard error (SE) for pairwise comparison of the 24 combinations of variety and environment is provided*.

a *For each trait, mean values for combinations of Variety, and Environment separated by different letters are significant (P < 0.05) according to the Student-Newman-Keuls multiple range test. When a mean is followed by four or more letters, the range of letters is indicated*.

### Variation for Chemical Composition Descriptors

Fruits from GH conditions had higher contents of glucose (on average 49%) than those from open field (Table 6). This higher content under GH conditions occurred in all varieties, except “Mallorquín.” The range of variation under OF went from 11.6 g/kg in “BGIB-018” to 21.8 g/kg in “Mallorquín,” while under GH went from 17.7 g/kg in “Domingo” to 31.5 g/kg in “Punteta.” A similar situation to that of glucose occurred for fructose content, with higher values (37.9% on average) under GH conditions, except for “Mallorquín.” A wide variation was observed among varieties for fructose content, in particular under OF, with values ranging from 4.2 g/kg for “Estrella” to 19.7 g/kg for “Moradeta,” while for GH values ranged between 13.2 g/kg for “Palamós F1” and 22.7 g/kg for “BGIB-018” (Table 6). Differences among environments for average values of citric acid content were non-significant, although many differences among environments were observed for individual varieties. In this respect, the ranges of variation under OF went from 1.53 g/kg in “Mallorquín” to 5.82 g/kg in “SEL1,” while under GH went from 2.31 g/kg in “Domingo” to 9.46 g/kg in “BGIB-198” (Table 6). Ascorbic acid content was higher under GH than under OF (on average 4.4%), although for three varieties, values were higher under OF. The variety with lowest values under both conditions was “BGIB-107” with values of 277 mg/kg and 301 mg/kg under OF and GH, respectively, while the one with highest values was “Punteta,” with values of 393 and 420 mg/kg under OF and GH, respectively (Table 6). Lycopene contents were, on average higher (67.4%) under OF than under GH, although for “Palamós F1” and “Punteta,” higher values were obtained under GH. Considerable variation was observed for lycopene content in both environments with ranges between 13.8 mg/kg for “Estrella” and 70.7 mg/kg for “Moradeta” under OF, and between 9.5 mg/kg for “BGIB-107” and 25.7 mg/kg for “Punteta” under GH (Table 6). Similarly to lycopene, β-carotene contents were higher under OF (on average 17.2%) than under GH, except for three varieties. Ranges of variation for β-carotene varied between 6.8 mg/kg for “Manacor F1” and 13.0 mg/kg for “Domingo” under OF, and between 5.3 mg/kg for “BGIB-107” and 16.1 mg/kg for “Palamós F1” under OF (Table 6).

**Table 6:** Mean values for chemical composition descriptors for 12 long shelf-life tomato varieties grown in open field (OF) and greenhouse (GH) environments.

	**Glucose (g/kg)**^**a**^		**Fructose (g/kg)**		**Citric acid (g/kg)**		**Ascorbic acid (mg/kg)**		**Lycopene (mg/kg)**		β**-carotene (mg/kg)**	
**Variety**	**OF**	**GH**	**OF**	**GH**	**OF**	**GH**	**OF**	**GH**	**OF**	**GH**	**OF**	**GH**
BGIB-018	11.6 a	22.9 def	11.6 ab	22.7 b	4.80 ab	3.55 a	348 bcd	345 bcd	36.6 a	15.4 a	11.6 abc	6.2 a
BGIB-107	13.4 ab	23.1 def	19.6 b	21.4 b	3.65 a	4.27 a	277 a	301 ab	38.5 a	9.5 a	8.7 abc	5.3 a
BGIB-198	17.7 a–e	27.3 fg	10.7 ab	18.0 b	5.06 ab	9.46 c	325 a–d	340 bcd	32.3 a	14.1 a	9.3 abc	8.5 abc
Domingo	14.8 abc	17.7 a–e	12.0 ab	16.0 ab	3.85 a	2.31 a	342 bcd	379 c–e	22.0 a	18.8 a	13.0 abc	9.6 abc
Estrella	13.4 ab	23.1 def	4.2 a	19.2 b	5.19 ab	3.06 a	336 bcd	369 c–e	13.8 a	12.6 a	11.6 abc	8.7 abc
Mallorquín	21.8 c–f	20.0 b–f	19.0 b	18.0 b	1.53 a	4.86 ab	367 b–e	377 c–e	26.1 a	19.6 a	7.7 ab	7.9 ab
Manacor F1	15.5 a–d	23.5 ef	9.3 ab	15.7 ab	5.05 ab	2.96 a	345 bcd	378 c–e	20.8 a	18.3 a	6.8 ab	8.7 abc
Moradeta	20.8 b–f	25.6 ef	19.7 b	15.0 ab	2.43 ab	2.44 a	320 abc	338 bcd	70.7 b	23.7 a	15.0 bc	7.7 ab
Palamós F1	17.5 a–e	23.7 ef	11.5 ab	13.2 ab	5.23 ab	8.78 bc	358 bcd	328 a–d	22.9 a	25.5 a	7.7 ab	16.1 c
Punteta	14.3 abc	31.5 g	4.7 a	13.9 ab	4.35 a	2.57 a	393 de	420 e	20.7 a	25.7 a	11.9 abc	9.9 abc
SEL1	17.2 a–e	24.2 ef	11.3 ab	14.1 ab	5.82 ab	2.88 a	343 bcd	372 c–e	32.2 a	22.4 a	10.7 abc	9.1 abc
UIB-2-70	14.9 abc	24.8 ef	15.1 ab	17.6 b	5.25 ab	6.09 ab	356 bcd	354 bcd	32.6 a	15.2 a	8.0 ab	7.6 ab
SE	1.7		2.6		0.92		13		6.6		1.6	

*The significance of the effects Variety, Environment (OF vs. GH), and Variety × Environment are presented in Table 2. The standard error (SE) for pairwise comparison of the 24 combinations of variety and environment is provided*.

a *For each trait, mean values for combinations of Variety, and Environment separated by different letters are significant (P < 0.05) according to the Student-Newman-Keuls multiple range test. When a mean is followed by four or more letters, the range of letters is indicated*.

### Principal Components Analysis

The first and second principal components in the PCA analysis accounted for 24.3 and 13.9% of the total variation, respectively (Table 7). The first principal component was positively correlated with several descriptors that had higher values under the OF environment, such as Foliage density, Style position, Style hairiness, Fruit width, Easiness of fruit to detach from pedicel, Fruit shoulder shape, Width of pedicel scar, and Yield (Tables 3, 4), and negatively to descriptors that had lower values under OF environment such as Leaf attitude, Pedicel length, Pedicel length from abscission layer, Fruit blossom end shape, Fruit shape, Daily moisture loss, Dry matter, Soluble solids, Glucose, and Ascorbic acid, but also with Color difference with true red and Taste index (Table 7) which although had higher values under OF, the relative differences between both environments were small (Tables 3–6). The second principal component (Table 7) was positively correlated with several descriptors that had lower values under the OF environment such as Inflorescence type, Number of flowers per inflorescence, Pedicel length, Size of corky area around pedicel scar, and Fructose (Tables 3, 6), and negatively with descriptors that had higher values under the OF environment such as Plant size, Degree of leaf dissection, Fruit size homogeneity, Fruit firmness, Lycopene, and β-carotene (Tables 3, 4, 6), but also to Plant growth type, Titratable acidity, Taste index, for which no significant differences existed between environments (Tables 3, 5), or to Soluble solids, which although had higher values under GH the relative differences among environments were small (Table 5).

**Table 7:** Correlation coefficients between morphological, agronomic, chemical properties, and chemical composition descriptors and first and second principal components obtained from a multivariate principal components analysis.

**Descriptors**	**First principal component**	**Second principal component**
**MORPHOLOGICAL DESCRIPTORS**		
Plant growth type	−0.070	−**0.155**
Plant size	0.047	−**0.271**
Stem pubescence intensity	0.060	−0.013
Foliage density	**0.208**	−0.101
Number of leaves under 1st inflorescence	−0.096	−0.074
Leaf attitude	−**0.176**	0.128
Degree of leaf dissection	0.092	−**0.187**
Anthocyanin coloration of leaf veins	0.008	0.130
Inflorescence type	−0.064	**0.271**
Number of flowers per inflorescence	−0.058	**0.151**
Style position	**0.181**	0.093
Style hairiness	**0.198**	0.014
Exterior color of immature fruit	0.076	−0.037
Presence of green (shoulder) trips on the fruit	−0.035	0.104
Intensity of greenback (shoulder)	−0.002	0.056
Fruit size homogeneity	−0.050	−**0.216**
Fruit length	0.076	0.091
Fruit width	**0.244**	0.096
Easiness of fruit to detach from pedicel	**0.166**	−0.052
Fruit shoulder shape	**0.240**	−0.019
Pedicel length	−**0.223**	**0.175**
Pedicel length from abscission layer	−**0.220**	−0.061
Width of pedicel scar	**0.204**	−0.135
Size of corky area around pedicel scar	−0.061	**0.276**
Skin color of ripe fruit	−0.123	−0.127
Fruit blossom end shape	−**0.223**	−0.149
Radial cracking	−0.016	0.078
Fruit fasciation	−0.006	0.057
**AGRONOMIC DESCRIPTORS**		
Fruit weight (g)	**0.240**	0.038
Fruit shape	−**0.204**	−0.050
Fruit firmness (Shore A standard units)	0.079	−**0.221**
Color difference with true red	−**0.208**	0.087
Yield (kg/plant)	**0.219**	−0.052
Daily moisture loss (%)	−**0.208**	0.087
**CHEMICAL PROPERTIES DESCRIPTORS**		
Dry matter (%)	−**0.213**	−0.149
Soluble solids (%)	−**0.192**	−**0.222**
pH	−0.076	−**0.199**
Titratable acidity (%)	0.047	0.070
Taste index	−**0.163**	−**0.286**
Antioxidant activity (mM TE/g)	−0.132	0.022
**CHEMICAL COMPOSITION DESCRIPTORS**		
Glucose (g/kg)	−**0.213**	0.124
Fructose (g/kg)	−0.028	**0.291**
Citric acid (g/kg)	0.039	0.045
Ascorbic acid (mg/kg)	−**0.152**	−0.092
Lycopene (mg/kg)	0.063	−**0.187**
β-carotene (mg/kg)	−0.015	−**0.198**
Variance explained (%)	24.3	13.9

*Correlation values with absolute values ≥0.15 are presented in bold font*.

**Figure 3:**
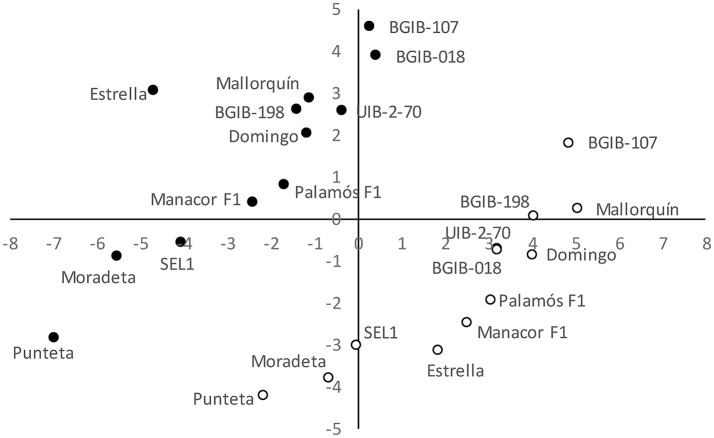
First (*x*-axis) and second (y-axis) principal components scatterplot, based on 46 variable descriptors (28 morphological,6 agronomic, 6 chemical properties, and 6 chemical composition) in 12 long shelf-life tomato varieties grown under open field (OF; open circles) and greenhouse (GH; solid circles) environments. The first and second principal components account for 24.3 and 13.9% of the total variation. Each variety is indicated by its code.

The projection of the 12 accessions grown in the OF and GH environments in the PCA plot clearly reveals a separation between both environments (Figure 3). Accessions grown under OF conditions plot in a diagonal area of the graph that spans values going from a combination of intermediate values for the first component and low ones for the second component to a combination of high values for the first component and intermediate ones for the second component (Figure 3). Regarding accessions grown under GH conditions they also plot in a diagonal area of the graph with comparatively lower values for the first component and higher ones for the second. The PCA plot reveals that within each of the environments, accessions plot in analogous areas of the of the scatterplot. Accessions with lowest values for first and second components under OF conditions (“Punteta,” “Moradeta,” and “SEL1”) are also the ones with lowest values for these components under GH conditions. The same occurs with accessions having highest values for both components, or intermediate values (Figure 3). Under both conditions accessions of the same origin plot in similar areas of the PCA graph. For example, in each of the environments, the three varieties from the “Tomata de Penjar” Quality Mark plot together and the same occurs for the four varieties from the Balearic Islands. Each of the two commercial selections plot together, and the same occurs for the two commercial hybrids (Figure 3).

## Discussion

Traditional long shelf-life tomato varieties carrying the *alc* mutation are well-adapted to open field cultivation and have specific characteristics that make them of special interest, like their tolerance to drought, extended post-harvest conservation period without refrigeration, and high contents in soluble solids (Mutschler et al., 1992; Conesa et al., 2014; Figàs et al., 2015b; Fullana-Pericàs et al., 2017, 2018). The two types of cultivation environment (OF and GH) are very different and our study was aimed at evaluating the performance of the tomatoes carrying the *alc* mutation under these two contrasting cultivation environments, which present many differences from the agronomic and management points of view, apart from taking place in different seasons of the year (Csizinsky, 2005; Peet and Welles, 2005; Figàs et al., 2018). As a consequence, the physiological mechanisms for growth and development processes acting in OF or GH conditions may be different, due to the great differences in temperature, solar radiation, wind, air humidity, and agricultural practices, among others (Tardieu, 2013).

Several descriptors that were uniform across the long shelf-life accessions and cultivation environments correspond to traits of taxonomic interest that distinguish tomato from some wild relatives, like the anther dehiscence type or the presence of fruit pubescence (Peralta et al., 2008), or traits that were introgressed from wild species into some modern tomato cultivars, such as the presence of jointless pedicel (Rick, 1967), or that appear as a physiological disorder caused by environmental factors or inappropriate cultivation practices, like the appearance of concentric cracking (Pascual et al., 2000). The two plant traits for which all the variation observed was caused by the enviromental effect (Plant growth type and Skin color of the ripe fruit) are monogenic and have a high penetration and expressivity (Carmen-Goren et al., 2003; Ballester et al., 2010), and confirm that there are *alc* long shelf-life varieties with determinate growth and that have colorless skin (i.e., resulting in pink colored fruits). The fact that the varietal effect was, in general, the largest one for morphological descriptors is important, as morphological descriptors used for characterizations should have a low environmental influence (Figàs et al., 2018). For the rest of descriptors, with the exception of fruit weight and fruit shape, which are largely genetically regulated (Panthee et al., 2013; El-Gabry et al., 2014; Monforte et al., 2014), as well as for dry matter, soluble solids and taste index, the cultivation environment, variety × environment, or residual effects were the most important contributors. In other works, it has been found that environmental effects together with their interaction with variety have a large effect on these traits in tomato (Kuti and Konuru, 2005; Ortiz et al., 2007; Roselló et al., 2010; Adalid et al., 2012; Panthee et al., 2013; Figàs et al., 2018).

The characterization of the different types of descriptors revealed that a high diversity exists among the different materials of *alc* long shelf-life tomatoes, as for most of the descriptors a wide range of variation was observed. This is in agreement with other works (Cebolla-Cornejo et al., 2013; Bota et al., 2014; Mercati et al., 2015), which have found high diversity for morphological and agronomic descriptors, molecular markers, and chemical properties and composition traits in this varietal type. This suggests that the genetic background of *alc* tomatoes is large and that there are ample opportunities for selection within this varietal type.

The cultivation environment had a significant effect for many morphological traits, which was expected, due to the great differences among OF and GH environments for tomato cultivation (Csizinsky, 2005; Peet and Welles, 2005; Figàs et al., 2018). The highest yield under OF conditions probably is a consequence of the higher irradiation and higher temperatures during the summer season, which favor yield in tomato, compared to suboptimal conditions in the greenhouse. Although the yields of tomato in greenhouse are often higher than in the open field, due to a more controlled environment (Csizinsky, 2005; Peet and Welles, 2005), long shelf-life tomato landraces evolved and were selected for open field cultivation and need high temperatures and radiation for optimal flowering (Mercati et al., 2015; Fullana-Pericàs et al., 2018), which probably accounts for the generally lower yields under greenhouse. Some selections and commercial hybrids, like “Domingo,” “Mallorquín,” and “Manacor” gave the highest yield under GH conditions and therefore may be recommended under these conditions. Among the traits affected, fruits from GH cultivation were easier to detach from the pedicel than those from OF. This is important in this varietal group, as fruits are on many occasions threaded in strings (Casals et al., 2012; Mercati et al., 2015) and berries have to be firmly attached to the pedicel to avoid fruits breaking off to the ground. Therefore, varieties grown under GH conditions might be less appropriate for being threaded than those from the OF. Fruits from GH are more pointed than those of OF. Pointed fruits can be a disadvantage of GH cultivation, as this characteristic may increase the risk of fruit damage and bruising during harvesting and handling. Nonetheless, some long shelf-life varieties (like “Punteta”) have pointed fruits. Greater fruit fasciation, an unfavorable trait, under GH might be caused by suboptimal environmental conditions resulting in fasciated flowers (Adams et al., 2001).

Fruits of *alc* long shelf-life tomato were relatively small when compared with other traditional tomato varieties (Bota et al., 2014; Figàs et al., 2015a) This is probably due to the negative correlation between fruit weight and post-harvest shelf-life in this varietal type (Casals et al., 2012). The fact that fruits from OF were larger than those of the GH could mean that the former could be less appropriate for post-harvest conservation. However, since OF fruits are generally more firm than those of GH indicates that the negative impact on post-harvest conservation of the larger fruit size of OF fruits can be compensated by their higher firmness. The fact that in most cases higher yields were obtained in the OF than under GH suggests a better adaptation of this varietal type to the traditional OF conditions, where it evolved and was selected (Casals et al., 2012; Cebolla-Cornejo et al., 2013; Bota et al., 2014; Mercati et al., 2015). Regarding post-harvest weight loss, it was low compared to standard tomato varieties (Javanmardi and Kubota, 2006; Pagno et al., 2017), and it was higher in fruits grown in GH, which is an indication of a better post-harvest performance of OF fruits.

The dry matter and soluble solids content was high compared to most standard tomato varieties (Rodríguez-Burruezo et al., 2005; Panthee et al., 2013; Figàs et al., 2015b). We found values of almost 8% for soluble solids in some varieties, suggesting that these materials could be a source of variation for breeding for dry matter and soluble solids content. Dry matter and soluble solids values have been higher under GH conditions, which probably is related to the reduced yield under these conditions. Several works indicate that in tomato there is a negative correlation between yield and soluble solids content (Dumas et al., 1994; Favati et al., 2009). pH and titratable acidity values were similar to those found in other works (Rodríguez-Burruezo et al., 2005; Panthee et al., 2013; Figàs et al., 2015b; Sánchez-González et al., 2015). In most *alc* long shelf-life varieties taste index was considerably higher than 1, which is considered as the optimal value for an equilibrated taste for salad tomato (Navez et al., 1999), and suggesting that fruits have an excess of soluble solids. Figàs et al. (2015b) also found that this varietal type, in general, has taste index values higher than 1. Traditional long shelf-life tomatoes carrying the *alc* mutation are generally used in a different way than the standard salad tomato (Casals et al., 2012; Romero del Castillo et al., 2014) and in most cases are used for rubbing into bread or used for cooking. Therefore, the different uses, compared to standard tomato used for being consumed raw in salads, probably have led to a selection of fruits with higher taste index in this varietal type. The fact that the antioxidant activity under GH conditions has been higher than under OF may be relevant for consumers (Diamanti et al., 2011), and the higher antioxidant activity might contribute to an extended post-harvest life (Zhang et al., 2013).

The levels of the chemical compounds analyzed here are similar to those obtained in other works for tomato in general (Rodríguez-Burruezo et al., 2005; Galiana-Balaguer et al., 2006; Panthee et al., 2013; Figàs et al., 2015b; Sánchez-González et al., 2015) and for this particular varietal type (Casals Missio et al., 2015; Figàs et al., 2015b), and reveal a considerable variation in the materials evaluated. As occurred for dry matter and soluble solids content, the average glucose and fructose levels were higher under GH conditions, which was expected, as sugars are a major constituent of soluble solids in tomato (Beckles, 2012; Figàs et al., 2015b). In the same way, as observed for titratable acidity, no differences in average values were observed for citric acid, the major organic acid in tomato (Galiana-Balaguer et al., 2006). Like antioxidant activity, ascorbic acid content was higher under GH conditions, although similarly to what was found for cherry tomatoes, lycopene levels were higher under OF conditions (Kuti and Konuru, 2005). Given the much higher levels of ascorbic acid than those of carotenoids, our results provide an indication that in tomato ascorbic acid may have a greater contribution than lycopene to the total antioxidant activity of Mediterranean traditional long shelf-life tomato varieties (Cano et al., 2003; Sánchez-Moreno et al., 2006; Figàs et al., 2015b). The fact that the norm of reaction for antioxidant compounds against the cultivation environment of the varieties tested was very different, so that some varieties had higher levels of the antioxidant compounds under GH than under OF, indicates that the G × E interaction can be exploited for long shelf-life materials with higher levels of antioxidants in either OF or GH conditions.

The PCA analysis clearly separated the combinations of variety and cultivation environment according to the cultivation environments. In a former work (Figàs et al., 2018), in which several varietal types were evaluated, we found that the PCA grouped the accessions according to varietal group and not to cultivation environment. However, within varietal group such distinction was unclear (Figàs et al., 2018). In our case, in which all materials belong to a single cultivar group, the clear separation for environment in the PCA indicates a major impact of the cultivation environment (open field vs. greenhouse) on characteristics of the plants and fruits of this varietal type (Csizinsky, 2005; Peet and Welles, 2005; Figàs et al., 2018). However, it is evident from the PCA that the distribution of accessions under OF or GH conditions follow a similar pattern indicating a good correlation of the global characteristics of individual varieties in different environments. The fact that individual varieties from each origin or varietal type cluster in the same area of the plot relative to other varieties in both OF and GH conditions reveal that a phenotypic differentiation may exist within this varietal group, which may be exploited for selection and breeding (Panthee et al., 2013; Scott et al., 2013). Importantly, the commercial selections and hybrids carrying the *alc* allele are not in the extremes of distribution of the PCA scatterplots for either OF or GH, revealing that they have similar characteristics to those of the landraces.

As observed in other works (Casals et al., 2012; Cebolla-Cornejo et al., 2013; Bota et al., 2014; Figàs et al., 2015a,b; Mercati et al., 2015), our results reveal that a large diversity exists in the traditional long shelf-life tomato varietal group characterized by carrying the *alc* allele, with the largest diversity being present in the landraces. Compared to the traditional OF cultivation of the landraces of this varietal group, cultivation under greenhouse had a high impact on morphological, agronomic, chemical properties and chemical composition. Generally GH cultivation had a negative impact on some morphological traits, like a greater easiness of fruit to detach from pedicel, which is important for the traditional threading of the fruits in strings for hanging (Casals et al., 2012), in productive traits (e.g., lower yield and firmness and higher post-harvest loss), and in lycopene and β-carotene contents. However, it also increased dry matter, soluble solids, antioxidant activity, and glucose, fructose, and ascorbic acid contents. Although large G × E interaction could be exploited for selection for adaptation to greenhouse of this varietal type (Scott et al., 2013), our results suggest that specific breeding programmes for selecting long shelf-life materials carrying the *alc* mutation of the traditional “de colgar,” “de penjar,” “de ramellet,” or “da serbo” specifically adapted to greenhouse cultivation are needed. In this respect, the diversity present in the landraces will be of great relevance for developing this new generation of varieties. Until these varieties are obtained, the evaluation of landraces and commercial selections and hybrids may allow identifying materials with better characteristics for greenhouse cultivation.

## Data Availability Statement

The raw data supporting the conclusions of this manuscript will be made available by the authors, without undue reservation, to any qualified researcher.

## Author Contributions

JP, MR, and SS planned the study. JP, MP, and SS supervised the research. MF, LP-D, CC, MG-M, and ES performed the morphological, agronomic, and chemical properties characterization. MR, MG-M, and ES performed the chemical composition characterization. MF, CC, and ES supervised the crops. MF and MG-C curated the data. LP-D, ER, and MP performed the statistical analyses. MF, JP, MP, and SS drafted the manuscript.

### Conflict of Interest Statement

The authors declare that the research was conducted in the absence of any commercial or financial relationships that could be construed as a potential conflict of interest.
